# Translational gut microbiome research for strategies to improve beef cattle production sustainability and meat quality

**DOI:** 10.5713/ab.23.0387

**Published:** 2023-12-29

**Authors:** Yasushi Mizoguchi, Le Luo Guan

**Affiliations:** 1 School of Agriculture, Meiji University, Tama-ku, Kawasaki, Kanagawa 214-8571, Japan; 2 Department of Agricultural, Food and Nutritional Science, University of Alberta, Edmonton, Alberta, T6G 2P5, Canada; 3 Faculty of Land and Food Systems, the University of British Columbia, Vancouver, British Columbia, V6T 1Z4, Canada

**Keywords:** Beef Cattle, Feed Efficiency, Meat Quality, Methane Emission, Rumen/Gut Microbiome

## Abstract

Advanced and innovative breeding and management of meat-producing animals are needed to address the global food security and sustainability challenges. Beef production is an important industry for securing animal protein resources in the world and meat quality significantly contributes to the economic values and human needs. Improvement of cattle feed efficiency has become an urgent task as it can lower the environmental burden of methane gas emissions and the reduce the consumption of human edible cereal grains. Cattle depend on their symbiotic microbiome and its activity in the rumen and gut to maintain growth and health. Recent developments in high-throughput omics analysis (metagenome, metatranscriptome, metabolome, metaproteome and so on) have made it possible to comprehensively analyze microbiome, hosts and their interactions and to define their roles in affecting cattle biology. In this review, we focus on the relationships among gut microbiome and beef meat quality, feed efficiency, methane emission as well as host genetics in beef cattle, aiming to determine the current knowledge gaps for the development of the strategies to improve the sustainability of beef production.

## INTRODUCTION

Among United Nations Summit’s 17 goals for the Sustainable Development Goals proposed in September 2015 [1], “Zero Hunger”, “Good Health and Well-being”, “Climate Action”, “Life below Water”, “Life on Land” are relevant to livestock production. In order to achieve those goals with increasing human populations, sustainable husbandry of livestock animals is essential. Ruminants, unlike monogastric animals, have the ability to break down human inedible plants/food to produce beneficial nutrients and provide animal protein resources such as meat and milk to humans. Among them, cattle are a valuable animal species of animal protein resources, and its global population reached 1,529 million in 2021 [2]. Global meat production approached 360 million tons in 2022, increased 1.2% from 2021 with beef production reached 73.9 million tons [3]. However, cattle produce enteric methane, one of major greenhouse gases, consume human edible cereal grains to achieve high productivity, and require/occupy arable lands. Therefore, the improvement in cattle production efficiently is essential to improve the sustainability of the cattle production.

Recent breakthroughs in high-throughput omics technologies (Metataxonomics, metagenomics, metatranscriptomics, metaproteomics, and metabolomics) have enabled the comprehensive understanding of rumen and gut microbiota and its interactions with host animals, leading to the identified linkage between the rumen/gut microbiome and many cattle production traits such as feed efficiency and methane emission. However, the translational strategies from the obtained tremendous omics data are still lacking due to the complexity of the rumen microbiome and challenges to interpret the omics data for the causality. Microorganisms that live in the gastrointestinal tract (GIT) of ruminants have a mutualistic relationship with the host that affects the growth and health of the ruminants. These microorganisms produce the metabolites (such as short chain fatty acids [SCFA]) and microbial proteins necessary for cattle’s growth and productivity. Although the rumen/gut microbiome has been linked to feed efficiency and methane emission traits, the knowledge on its impact on the meat quality of beef is not well defined. In the beef industry, carcass (carcass weight, rib eye area, backfat thickness, meat yield, and marbling etc.) and meat quality (shear force, pH, myoglobin content, meat redness, intramuscular fat content, fatty acid (FA) composition, etc.) are important traits that affect product prices, human health and consumers’ choice (e.g. [4]). It has been reported that the gut microbiome of pigs and chickens can affect the meat quality traits (e.g. [5–8]). However, there is little knowledge about the relationship between the meat quality, host genetics and microbiome in ruminants and how such relationships could be affected by other production traits. Therefore, this review focus on recent research on gut (rumen and lower gut) microbiome, metabolites, host genetics and meat quality as well as production traits (feed efficiency and methane emission) in beef cattle, with the aim to seek scientific foundations for novel strategies to improve beef quality and sustainability of cattle production through gut microbiome interventions.

## EFFECT OF HOST GENETICS ON RUMEN MICROBIOME IN BEEF CATTLE

Recent studies have revealed the rumen microbiome is influenced by host genetic factors, highlighting genetics are involved in the determination of colonization of microbes in the rumen. Several studies have reported breed effect on the composition of rumen microbiota [9,10]. For example, the Firmicutes/Bacteroidetes (F/B) ratio in the rumen of Angus (n = 20, male) was 0.65, while it was 4.49 in Chinese Simmental (n = 20, male) [10]. Similarly, the abundance of twenty-five bacterial species and 10 microbial functions were different between Black and Red Angus [11], suggesting the roles of host in affecting rumen bacterial colonization. Recent research further revealed that some ruminal microbes are heritable and also identified regions on the genome are associated with the abundance of bacterial taxa in the rumen of beef cattle. The heritability of bacteria’s total bacterial abundance (estimated by the 16S rRNA gene copy number) was 0.16 (709 cattle) demonstrating that rumen microbes and their fermentation products such as volatile fatty acids (VFAs) can be affected by host genetic features [9]. Approximately 30% of the rumen microbial taxa has been shown to be potentially hereditary to the host [9,10]. Cardinale and Kadarmideen [13] also showed heredity (h^2^>0.15) of 11 taxa in the rumen microbiome (1,016 dairy cow). Compared to members belong to Firmicutes, taxa belonging to Bacteroidetes have lower heritability and they are more responsive to the dietary changes [9,12].

Genome-wide association study has also identified genomic regions associated with the abundance of several rumen microorganisms such as genus *Succiniclasticum* was found to be associated on chromosome 1 and 2 and *Fibrobacter succinogenens* was associated with and chromosome 27 in cattle [14]. *Succiniclasticum ruminis* is a common rumen bacterium that specializes in converting succinate to propionate to produce energy [15] and *Fibrobacter succinogenens* has been characterized as one of the major cellulolytic microorganisms, helping to break down cellulose in the rumen [16]. These finding suggest that the host genome may regulate microorganisms involved in feed digestion and fermentation in the rumen. In addition, Li et al [9] identified 19 single nucleotide polymorphisms (SNPs) associated with 14 rumen microbial taxa and some of these SNPs were associated with the quantitative trait loci (QTL) of feed efficiency. These findings further suggest that it may be possible to identify host genetic factors that affect the rumen microbiome that can be used in future breeding strategies to improve rumen function and feed efficiency [12]. Recently, Zang et al [17] provided a comprehensive assessment of heritable and non-heritable ruminal microbiota and showed varied functions between them in the rumen dairy cow. This study indicated that the functions of heritable bacteria were mainly enriched with functions in FA, amino acid, and energy metabolism, while non-heritable bacteria were mainly enriched with functions in amino acid and ribonucleotide metabolism. These suggest that heritable bacteria play an important role in carbohydrate metabolism, the fundamental step in converting plant material to VFAs in the rumen.

Similar host genetic effect on rumen microbiota was also observed for the hindgut microbiota. Heritability of fecal microbes (*Oscillospira* [*h*^2^ = 0.46], *Sutterella* [*h*^2^ = 0.42], and *Roseburia* [*h*^2^ = 0.21]) were reported for Angus-Brahman multibreed cattle (226 preweaning calves, 176 calves and 105 fatting steers) and host SNPs were associated with the relative abundance of butyrate-producing bacteria [18]. Interestingly, the minor allele frequencies of SCFA receptors (*GPR43* and *GPR109A*) differed between breed composition groups [18]. Although a few studies have reported the breed effect on the fecal microbiota of calves [19] and differences in the composition and function of the fecal microbiota between Angus (n = 20, male) and Chinese Simmental cattle (n = 20, male) [10], the host genetic effects have been mainly reported for the rumen microbiome of cattle to date. It is known that whether the lower gut microbes could also be heritable because fecal microbiota does not represent the those in the small and large intestinal tracts.

Overall, the findings on heritable rumen microbes have provided new insights into the genetic interactions between microbiome and hosts. The heritability of rumen/gut microbes research is still in the infancy stage and only has been reported in “controlled” and small populations, the “true heritability” (which taxa can be passed to the offspring) and “breeding values” should be further assessed to determine causal and persistent microbes and microbial function, and the possibility of using genetic selection and breeding to manipulate desirable and efficient rumen microbiota.

## RECENT RESEARCH ON RUMEN MICROBIOME CONTRIBUTING TO MEAT QUALITY IN BEEF CATTLE

Many meat quality traits are genetically regulated, and this has been widely reported especially on gene maker identification and expression related to carcass traits and so on. (e.g. [20]). Marbling has a great influence on palatability, which depends on tenderness, juiciness and flavor [21], and is one of the major factors affecting the meat grade. The growth of muscle and adipose in beef cattle is important for improving meat quality. Accelerating adipogenic differentiation of fibrogenic/adipogenic progenitor cells in muscle increases intramuscular adipocytes, reduces connective tissue, and improves marbling and tenderness in beef meat [22]. Intramuscular fat deposition is determined by both genetic and environmental factors. Intramuscular adipocytes are formed by lipid filling of fibroblasts within the muscle perimeter and consist of adipocytes distributed between muscle fibers [23]. Intramuscular adipocytes are composed mainly of phospholipids and triglycerides, the content of which is determined primarily by number and size, and provide a “marbling” site for subsequent fat deposition [24]. It is known that marbling related traits are highly heritable. Quantitative trait loci for candidate genes associated with marbling, FA composition and carcass weight, etc. has been identified in Japanese Black cattle (e.g. [20]). The estimation of marbling score heritability was 0.34 to 0.68 in various breeds [25]. The same review also detailed genetic dynamics and molecular mechanisms that affect the marbling levels (intramuscular fat deposition) (e.g. [25]). Although the genetic effects have been identified for the meat quality related traits, recent findings of gut microbiome’s contribution to fat metabolism in animals suggest the interplay between host genetics and microbiome could also influence the meat quality traits.

Recent studies have shown that rumen and gut microbiome can affect meat quality (e.g. [26–29]). Kim et al [26] revealed a potential linkage between marbling and the rumen microbiome in Hanwoo steers. In their study, species richness in the rumen tended to be higher in the steers with high-marbling scores, and the overall rumen microbiota was different between the low-marbling score and high-marbling score groups. They also reported that RFP12, *Verrucomicrobia*, *Oscillospira*, *Porphyromonadaceae* and *Paludibacteri* were more abundant in the high-marbling score group, while *Olsenella* was more abundant in the low-marbling score group. Several marbling-related bacterial taxa also contributed to the enrichment of two lipid metabolism pathways including “α-linolenic acid metabolism” and “FA biosynthesis” in the high-marbling score associated microbiome [26]. In another study by Krause et al [27], it is shown that relative abundances of bacterial family S24–7 family and *Allerminsia*, *Blautia*, *Klebsiella*, *Peptostreptococcus*, *Slenimonas* genera in rumen were positively correlated with marbling score in Angus steers. The crude fat content of muscles tended to be associated with the increased ratio of *Firmicutes* to *Bacteriodetes* in Simmental crossbred finishing steers [28]. In lambs, significantly higher relative abundance of *Fibrobacter* and *Succinivibrio* were associated with higher intramuscular fat in the group fed with a lower Alfalfa content ratio, and had a high content of palmitic, stearic, elaidic, and alpha-linolenic in *longissimus lumborum* muscle [29]. The same study also showed that the relative abundance of both *Prevotellaceae_UCG_003* and *Prevotellaceae_UCG_004* was positively correlated with the C18:1 cis-9 and negatively correlated with the C18:0. In addition, it has been reported that the relative abundance *Proteobacteria* was negatively correlated with C18:0 in *longissimus thoracis et lumborum* [30]. A recent study by Zhang et al [31] found that rumen *Selenomonas 1* and *Christensenellaceae R-7 group* was positively correlated and n-6 polyunsaturated fatty acids (PUFAs), and n-6/n-3 ratio, respectively in *Longissimus lumborum* of Black Tibetan sheep. The same study also identified *Rikenellaceae RC9 gut group* and *Prevotella 1* were negatively associated with n-6/n-3 ratio, whereas *Lactobacillus* was positively associated with n-3 PUFA and C16:0/C18:1 ratio as well as *Eubacterium coprostanoligenes group*, *Quinella* and *Christensenellaceae R-7 groups* were negatively associated with n-3 PUFAs [31]. In addition to the fat acid profiles, the same author also reported *Christensenellaceae R-7 group* was positively correlated with leucine, isoleucine, and valine and *Rikenellaceae RC9 gut group*, *Prevotella 1* and *Lactobacillus* in rumen were negatively correlated with arginine, phenylalanine, leucine, isoleucine, proline, valine, threonine, asparagine, tryptophan, taurine and total essential amino acid content in meat (*Longissimus lumborum*) [31]. These microorganisms are involved in the deposition of amino acids and FAs and produce various metabolites (maltotriose, pyruvate, L-ascorbic acid, chenodeoxycholate, D-glucose 6-phosphate, glutathione) involved in the regulation of meat quality [31]. Additionally, Li et al [32] revealed microbiota of the rumen, duodenum and colon were associated with FA content in sheep muscle. Table 1 summarizes the relationship between microbiome and meat quality. These findings suggest that rumen microbes could affect meat quality through affecting muscle growth and adipogenesis.

## RECENT RESEARCH ON LOWER GUT MICROBIOME CONTRIBUTING TO MEAT QUALITY IN BEEF CATTLE

Recent study reported that the F/B ratio was higher in feces of feedlot-fed Angus cattle than that in grazing cattle [33], indicating that the lower gut microbiota may influence the meat quality of cattle. In a recent study using Angus and Xinjiang brown cattle, intramuscular fat content was positively correlated with fecal *Prevotella copri*, *Blautia wexlerae*, and *Ruminococcus gnavus* bacterial species, and backfat thickness was negatively correlated with fecal *Blautia wexlerae* [34]. The relative abundance of unclassified Mogibacteriaceae and *Succiniclasticum* in the hindgut had a positive linear relationship with intramuscular fat content in the multibreed Angus-Brahman herd [35]. In a study on the relationship between gut microbiota and meat quality using feces and longissimus dorsi in Angus and Chinese Simmental, bacterial species such as *B. uniformis*, *B. vulgatus*, *R. inulinivorans*, *E. rectale*, *C. catus*, *F. prausnitzii* were positively correlated with expression of muscle metabolism-related genes, including ATPase sarcoplasmic/endoplasmic reticulum Ca2^+^ transporting 1 (*ATP2A1*), myostatin (*MSTN*), actinin alpha 3 (*ACTN3*), myosin light chain, phosphorylatable, fast skeletal muscle (*MYLPF*), myosin light chain 1 (*MYL1*), and troponin type 3 (*TNNT3*) [10]. Among these species, *R. inulinivorans*, *E. rectale*, and *C. catus* were highly abundant in Angus cattle, whereas *B. uniformis* and *B. vulgatus* were enriched in Chinese Simmental cattle. The muscle gene expressions and their functions of Angus cattle and Chinese Simmental cattle were significantly different [10], suggesting that both genetics and gut microbiome may influence the varied meat quality traits in these cattle.

As described above, many studies have only used fecal samples to assess the relationship between the lower gut microbiota and meat quality. It is known that the lower GIT consists of small intestine (duodenum, jejunum and ileum) and large intestine (cecum, colon and rectum) regions, where microbiota varies in composition depending on the intestinal regions [36]. A recent study showed that decreased testosterone by castration was associated with elevated branch-chain amino acids and *Peptostreptococcaceae* in the small intestine and elevated *Cellulolytic bacteria* in the large intestine, which could be associated with increase intramuscular fat in cattle [37]. Such microbiota shift could be associated with increased serum branched-chain amino acids (BCAAs) [37]. 3-Hydroxyisobutyrate, a catabolic intermediate of BCAA valine, activates endothelial FA transport, stimulates muscle FA uptake, and promotes muscle lipid accumulation [38]. These findings suggested that high levels of serum BCAAs in castrated cattle may contribute to intramuscular adipocyte accumulation [37]. Castration increases transcription levels of key genes encoding enzymes involved in the irreversible gluconeogenic reaction from pyruvate to glucose and enzymes involved in the uptake of glycogenic substrates and hepatic gluconeogenic gene expression levels were associated with intramuscular fat deposition [39]. In addition, cattle with myostatin gene mutation which negatively regulates muscle development was shown to affect the metabolism of *Ruminococcaceae_UCG-013*, *Clostridium_sensu_stricto_1* and *Ruminococcaceae_UCG-010* in the gut [40]. Myostatin-edited sheep increased abundance of *Firmicutes*, whereas decreased abundance of *Bacteroidota* [41], suggesting that muscle development and gut microbiota are closely involved.

Marbling traits are great economic value and a valuable biological phenotype. As discussed above, it has become clear that marbling involves various factors such as rumen and gut microbiome, metabolites and host genetic characteristics (Table 1), revealing the recent research on the relationship between gut microbiome and meat quality. It is noticeable that management practices (die, feed additives) can also affect the meat quality, and the diet can alter the gut microbiome. Future research taken into account of all these various factors is needed for future breeding and management programs to achieve the better meat quality.

## HOST AND MICROBIAL METABOLOME CONTRIBUTION TO BEEF MEAT QUALITY

Recent research has further revealed the metabolome of cattle could play an important role in affecting beef meat quality. Li et al [42] reported some plasma metabolites (3-hydroxybutyric acid, creatine, D-glucose, succinic acid and so on) were associated with various carcass traits (hot carcass weight, rib eye area, average backfat thickness, lean meat yield, carcass marbling score) and further identified candidate genes associated with metabolites. Incorporating metabolomics-related data may lead to improved genomic prediction accuracy as the metabolites can directly affect cattle metabolism [42]. Zhang et al [43] indicated there was a strong association between fat content and lipid (ether lipid, glycerol lipid and glycerophospholipid) and carbohydrate metabolism (carbohydrate digestion and absorption, sucrose and starch, and galactose) in black Tibetan sheep. Another recent study also revealed that intramuscular fat content was positively correlated with the metabolites including succinate, oxoglutaric acid, L-aspartic acid and L-glutamic acid, and negatively correlated with GABA, L-asparagine and fumaric acid and *Prevotella copri*, *Blautia wexlerae*, and *Ruminococcus gnavus* [34]. Backfat thickness was negatively correlated with the metabolites including succinate, L-aspartic acid and L-glutamic acid and positively correlated with GABA, L-asparagine and fumaric acid and *Blautia wexlerae* [34]. Metabolomic study of Japanese black beef suggested that several metabolites (decanoic acid, uric acid, elaidic acid, 3-phosphoglyceric acid) in meat were potential biomarkers of intramuscular fat to assess marbling levels [44]. A recent study identified fecal metabolites that influence marbling traits and demonstrated their potential as metabolic biomarkers [45]. Higher levels of tricarboxylic acid (TCA) cycle (cis-aconitic acid, citric acid, isocitric acid), lipid synthesis (3-hydroxybutiric acid, glycerol 3-phosphate), FA metabolism (o-acetylcarnitine), diabetes (methylhistidine, asymmetric dimethylarginine), and glucose homeostasis (hippuric acid) were found for blood metabolme in Wagyu cattle compared to Holstein [46]. Early castration of Holstein calves was shown to improve beef marbling grade [47]. Untargeted metabolomics analysis of the liver showed that the early castration group had increased betaine, glycerol 3-phosphate, glutathione, acetylcarnitine, riboflavin, and alanine and decreased diethanolamine, glycine, and 2-hydroxyglutarate, which were associated with the increased marbling [47]. The above findings suggest that both host and microbial metabolites could play a role in affecting meat quality. The relevance of metabolites in meat quality, especially in intramuscular adipogenesis, is influenced by a variety of environmental and genetic factors. It remains unclear how the microbiome is involved in these metabolites. Future research is needed to understand the role of microbial metabolites in affecting meat quality and whether these metabolites can be manipulated through microbiome interventions.

## LINKAGE AMONG RUMEN MICROBIOTA, RUMEN FATTY ACID PROFILES AND BEEF MEAT QUALITY

Short-chain fatty acids (mainly acetic acid, propionic acid, and butyric acid) are the main end products of rumen microbial fermentation. Acetate and glucose have been reported to be the major precursors of FA biosynthesis, with glucose preferred for intramuscular adipocytes and acetate preferred for subcutaneous fat [48–50]. It has been shown that acetate promotes lipogenesis more in the subcutaneous adipose tissue than that in the intramuscular adipose tissue in Wagyu and Angus steers [51]. In Wagyu crossbred steers, seven blood metabolites (3-hydroxybutyrate, propionate, acetate, creatine, histidine, valine, and isoleucine) were identified to be positively associated with marbling and the number of days the animals on a starch-rich diet [50]. The improved dietary energy increased propionate and intramuscular fat content of beef and decreased the acetate/propionate ratio could be related to the changes in the rumen microbiota.

High propionate and low butyrate levels were found in the rumen of Japanese black cattle during late fattening period when the high-energy diet prompted ruminal propionate production and hepatic gluconeogenesis, which may explain the elevated concentrations of blood lipid metabolites between 20 and 28 months of age [52]. In the same study, ketone concentrations decreased significantly during the middle and late fattening stages which may be due to reduced levels of its precursor, ruminal butyrate [52]. Similar to Simmental hybrid cattle, *Succinivibrionaceae_UCG-002* had a positive correlation with propionic acid and butyric acid and a negative correlation with rumen pH [53]. Propionate is converted to acetyl-CoA and enter the TCA cycle and is used for gluconeogenesis in the liver, where it can become a carbon donor in *de novo* FA synthesis [49]. Connolly et al [54] reported steers with high marbling at slaughter tended to have higher levels of propionate, 3-hydroxybutyrate, acetate, creatine, glucose, anserine, and arginine, but levels of lipid groups, choline and acetyl groups were low in blood.

Lower levels of total SCFAs and their major components, acetate, propionate and butyrate were observed in the ruminal fluid of gras-fed cattle compared to grain-fed cattle [55]. Grain-fed cattle had higher proportions of *Succinivibionaceae* and *Succinimonas*, starch-fermenting bacteria that produce succinate, acetate and lactate in the rumen. When the roughage content increased, marbling tended to decrease, and propionate in the rumen decreased linearly [56]. When acetate, propionate and glucose were injected into the rumen, the lipid content of longissimus thoracis muscle, intramuscular adipose tissue was more affected by propionate and glucose than acetate, while lipid content of subcutaneous adipose tissue did not change [57]. Maximum intramuscular adipocyte volume was highest when injected with propionate, and maximum glucose incorporation (both glyceride-FA and glyceride-glycerol) were observed [57]. In the future, it is necessary to elucidate more detailed physiological mechanisms of VFA and glucose that affect beef meat quality.

Additionally, conjugated linoleic acid (CLA) is known to be involved in anticarcinogenic, antiobesity, antidiabetic, antihypertensive properties, etc. and is mainly found in meat and dairy products derived from ruminant animals [58]. CLA, primarily C18:2 *cis*-9, *trans*-11 is biosynthesized by ruminal bacteria via isomerized C18 PUFAs and is known as an intermediate in the biohydrogenation process [58]. Subcutaneous fat had highest CLA levels among subcutaneous fat, kidney fat, intermuscular fat and intramuscular fat in Polish Holstein-Friesian and Limousin crossbred cattle [59,60]. Cattle with different breeds could also have different CLA contents in subcutaneous adipose tissue [61]. Martínez-Álvaro et al [62] developed microbiota-driven breeding strategies to improve beef quality with increased content of long-chain n-3 FAs (C18:3n-3, C20:5n-3, C22:5n-3, and C22: 6n-3), cis-9, trans-11 C18:2, and trans-11 C18:1, which is beneficial for human health. It has been reported *Megasphaera. elsdenii* can convert lactate into butyrate and propionate, reducing lactate accumulation and thereby increasing ruminal pH [63], which may affect the rumen environment for CLA biosynthesis. For example, vaccenic acid-producing bacteria (*Butyrivibrio fibrisolvens*) and ciliate protozoa, both increased ruminal content of *trans*-11 and *cis*-9, *trans*-11-CLA in the pH range between 5.6 and 6.3 [63]. It is speculated when pH is low, less trans-11 and cis-9, trans-11-CLA could be reduced. Yeast supplementation to ruminant diets positively alters rumen biohydrogenation pathways to synthesize more beneficial biohydrogenation intermediates (*trans*-11 and *cis*-9, *trans*-11) [63]. This suggests that more dietary sources of linoleic acid, linolenic acid, and oleic acid, along with beneficial biohydrogenation intermediates (*trans*-11 and *cis*-9, *trans*-11), can be absorbed in milk and meat [63]. From these findings, it suggests future research is needed to determine how ruminal and lower gut microbiome directly affect FAs profiles in the gut and tissues and their causal roles in affecting meat quality.

## ASSOCIATION BETWEEN BEEF MEAT QUALITY AND METHANE EMISSION

To date, there is little research into the relationship between methane emissions and meat quality, although it is important for the industry to know what are “trade-offs” between these two important traits.

There have been several studies on supplements that suppress methane emissions and meat quality in recent years. Studies of feeding red seaweed (*Asparagopsis taxiformis*) found that enteric methane emission was reduced, but there was no difference in average daily gain, carcass quality, strip loin proximate analysis, shear force or consumers’ taste preferences [64]. Nutrient treatments (nitrate and lipid) that reduced methane emissions also did not adversely affect meat qualities such as tenderness, juiciness and so on [65]. Similary, tannins, known to have antioxidant properties, reduced methane emissions in sheep, but had no effect on Bapedi ram meat sensory attributes [66]. Mulberry supplementation has been reported to affect the meat quality. Mulberry leaf powder supplementation improved the color (redness), tenderness, and water retention of the *longissimus lumborum* muscle in Hu lambs [67]. The mulberry and its extracts are known to improve immune function and suppress enteric methane production in ruminants [68], and it is speculated they could alter the rumen and gut microbiome which can impact on muscle growth and lipid profiles. Indeed, partial replacement of Chinese wild rye with mulberry leaves in the diet of sheep decreased saturated FA content and increased unsaturated FA content in *longissimus dorsi* muscle [69]. A meta-analysis on small ruminants showed that dietary supplementation with essential oil (EO) increased ruminal propionic acid concentration, reduced methane output, rumen ammonia nitrogen concentration, and the number of total protozoa and methanogens [70]. Furthermore, EO reduced the yellowness of small ruminant meat, suggesting that it improved the quality of fresh meat quality [70]. Dietary supplementation with sunflower seeds decreased methane production and increased the proportion of vaccenic and rumenic acids in intramuscular and subcutaneous fat [71]. Feed supplementation research on methane emissions and meat quality is still limited. Future development of omics-based microbiome research including metagenome/metatranscriptome and metabolome will enable to identify useful microbiota and microbial manipulation through feed additives and supplementation for improving meat quality and reducing methane emissions.

Recent studies also revealed that the host-genome is also involved in methane emissions [72]. Incorporation of methane emission indices into animal breeding programs has been recently discussed [73]. Mahala et al [73] summarized that the heritability of methane emission was moderate in many studies, ranging from 0.11 to 0.45. There are several researches on strategic approaches to genetically breeding cattle with reduced methane and improved meat quality. Rowe et al [74] demonstrated that the resulted physiological changes by implementing breeding as a mitigation strategy for low methane yield did not adversely affect meat quality or carcass characteristics in New Zealand sheep. Martínez-Álvaro et al [62] also included 31 microbial functions in a microbiome-driven breeding strategy specifically selected to increase healthy FA indicators in beef and reduce methane emissions. These suggest that genetic breeding could include index such as selected microbes and/or microbial functions/metabolites.

## ASSOCIATION AMONG RUMEN MICROBIOME, FEED EFFICIENCY AND BEEF MEAT QUALITY

Residual feed intake (RFI) is one of measures for cattle’s feed efficiency, referring the difference between actual feed intake and expected feed requirements for maintenance and weight gain, which is the main energy consumption of growing animals [12]. Animals with low RFIs are considered more efficient because they eat less than expected [12]. It has also been shown that cattle with higher feed efficiency have less methane emission [75]. Here, we will focus on the feed efficiency and meat quality traits.

There are several sheep research on the relationship between feed efficiency and meat quality. In lambs, animals with low RFI had higher shear forces [76], and higher cooking loss [77] than those with high RFI. The lambs fed forage rape had decreased the feed conversion ratio, increased average daily gain, intramuscular α-linolenic acid content, various amino acid contents in the muscle and relative abundance of cellulolytic bacteria and short-chain FA producing bacteria, including members of *Succiniclasticum*, *Fibrobacter*, and *Lachnospiraceae* in rumen [78]. Circulating leptin and insulin like growth factor 1 concentrations were higher in low-RFI or high-RFI, respectively. Variations in RFI did not affect redness or tenderness of meat [79].

Although studies showed that improved feed efficiency can be achieved without adversely affecting growth and carcass traits in Angus cattle [80], Zhou et al [61] found that low RFI steers had the lower proportion of beneficial FAs such as 18:2n-6, total n-6, PUFA, and t11-18:1 and c9, t11-CLA, as well as the higher unhealthy FAs (i.e. 16:0) in subcutaneous fat tissue. Recently, the relationship between marbling traits and feed efficiency has also been reported. In the marbling traits of Angus, steers bred from high-RFI from dam/sire were higher than low-RFI from dam/sire [81]. In Nellore steers, low RFI (feed efficient) animals have 11.8% lower intramuscular fat mass than that in high RFI (feed inefficient) animals [82]. However, Fedelis et al [83] reported no differences in carcass traits and meat quality in Nellore bulls between RFI groups. It has been reported that differences in RFI do not affect meat quality such as tenderness, juiciness, color and longissimus chemical composition in Nellore cattle [83]. Comparing feed efficiency and fat deposition, RFI was independently associated with subcutaneous fat, FA content, and intramuscular fat (IMF), and BTA20 was close to genes *CD180* and *MAST4* with respect to the association of IMF-RFI [84]. Recently, research of bacterial population in the duodenum, jejunum and ileum of Angus heifers [85] as well as study based on fecal samples of Angus steers [86] showed that differences in feed efficiency may be due to differences in gut microbial populations. However, there have been no reports on lower gut microbiota and meat quality in relation to feed efficiency.

Based on the estimated genetic parameters of Japanese Black steers, it was suggested that the selection of melting point or FA traits did not have a significant effect on feed efficiency [87]. The paper by Meale et al [81] concluded that: influence of selection for RFI of beef cattle did not substantially affect carcass quality as measured in offspring of Angus steers. However, fat and moisture content of particular muscles, muscle fibre type and area, plus more prominent differences in marbling were present [81]. If feed efficiency and meat quality could be improved at the same time, the significant benefits are expected in the beef cattle industry, highlighting the future need to research this area.

## CONCLUSION

**Figure 1:**
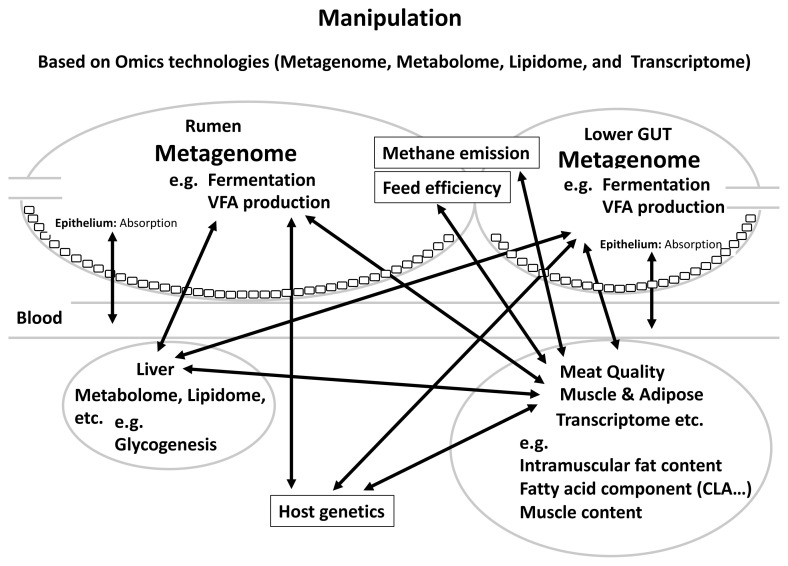
Schematic of the ruminal gastrointestinal manipulation regarding meat quality in beef cattle.

In this review, we mainly described the relationships between rumen and gut microbiome, feed efficiency, methanemission, host heritability and meat quality in beef cattle, revealing that gut microbiota and their metabolites together with host genetic factors and environmental factors such as diet can affect cattle production sustainability and meat quality. Figure 1 summarizes the proposed schematic of these relationships. We also assessed the importance of the relationship between CLA and content and microbiota in meat quality, which affects human health. Furthermore, by clarifying the relationship among methane emission, feed efficiency, meat quality, and gut microbiota, it is possible to improve these traits through manipulation rumen and lower gut microbiota: i) selection by breeding value evaluation; ii) manipulation of the GIT microbiota through feed additives; and iii) supplementation with probiotics, prebiotics and so on. It was considered that effective and efficient breeding manipulations are very important to accumulate further research and practice in the field of livestock farming. Although much evidence has revealed the important roles of gut microbes in cattle productivity and meat quality, it has not been possible to define the relationships between the symbiosis/interactions within microbiome such as bacteria, archaea, fungi and viruses and meat quality. In addition, no study yet has shown the relationship between small intestine microbiome and meat quality in cattle. It is critical to develop research methods/validation models to determine the cause and/or exact contribution of gut microbiome to meat quality, feed efficiency and methane emission. It is also noticeable that rumen epithelial tissue-attached microbiota differs from those associated with digesta. Tan et al [88] revealed higher active *Campylobacteraceae* and *Neisseriaceae* in the mucosal attached microbial community of low-RFI cattle and they are known to play a role as oxygen scavengers [88]. These suggest that rumen mucosal attached bacteria can also affect the rumen function such as epithelial nutrient absorption and pH homeostasis that directly affect cattle performance. There have been several reports on the relationship between rumen tissue gene expression and carcass traits. The transcriptome analysis of rumen tissue of Simmental cattle under different developmental stage revealed genes influencing carcass weight, stomach weight, marbling score, backfat thickness, ribeye area and lean meat weight [89]. It has become clear that host gene expression and epigenetic regulation could also influence the gut microbiota. However, the heritability and the roles of these microbes in affecting methane emission, meat quality are largely unknown, although there is evidence that they may have an impact on cattle feed efficiency. Future research using single-cell RNA-seq based transcriptomics may allow us to investigate the relevance of the development of mutualism between the rumen and its epithelial-attached microbes, in addition to it as a powerful tool to characterize the cell types and cellular functions in the rumen epithelial tissues [90]. Regardless, the information from this review has provided scientific bases for future novel microbiome-based solutions to enhance the production sustainability and improve the meat quality.

## Figures and Tables

**Table 1:** Summary of reported rumen/gut microbes associated with meat quality in beef cattle and sheep

Traits	Effect	Microbiome		Samples	Animals	Treatments	References
Marbling	Positive	Phylum	RFP12, Verrucomicrobia	Rumen	Hanwoo steers		[26]
		Genus	*Oscillospira, Paludibacter*				
		Family	Porphyromonadaceae				
	Nagative	Genus	*Olsenella*				
Marbling	Positive	Phylum	Verrucomicrobia, Rikenellaceae, S24–7, Verrucomicrobiaceae	Rumen	Angus steers		[27]
		Genus	*Akkermansia, Blautia, Klebsiella, Moryella, Peptostreptococcus, Selenomonas*				
	Negative	Phylum	Tenericutes, TM7, Erysipelotrichaceae, F16, RFN20				
		Genus	*Pseudomonas*				
Longissimus lipid content	Positive	Phylum	Actinobacteria, Verrucomicrobia, Coriobacteriaceae				
		Genus	*Dorea, Moryella*				
	Negative	Phylum	Cyanobacteria, Proteobacteria, Tenericutes, TM7, F16, *Succinivibrionaceae*, RFN20				
		Genus	*Pseudomonas, Succinivibrio*				
The concentration of caprylic acid in longissimus dorsi	Positive	Genus	*Moraxella, Riemerella*	Rumen	Simmental crossbred finishing steers	The effect of Herbal tea residue	[28]
The concentration of DHA in longissimus dorsi	Positive		*Moraxella, Riemerella*				
The concentration of DPA in longissimus dorsi	Positive		*Moraxella, Riemerella*				
The concentration of glucarate in longissimus dorsi	Positive		*Moraxella, Riemerella*				
The concentration of lauric acid in longissimus dorsi	Positive		*Moraxella, Riemerella*				
The concentration of linolenic acid in longissimus dorsi	Positive		*Acetitomaculum, Anaerovibrio, Anaerovorax, Blautia, Desulfovibrio, Howardella, Papillibacter, Schwartzia, Veillonellaceae*				
	Negative		*Riemerella*				
The concentration of phosphocholine in longissimus dorsi	Positive		*Anaerovibrio, Desulfovibrio, Olsenella, Papillibacter, Rikenellaceae, Schwartzia, Veillonellaceae*				
The concentration of G6P in longissimus dorsi	Positive		*Schwartzia, Succiniclasticum*				
Intramuscular fat in longissimus dorsi	Positive	Genus	*Prevotella copri, Blautia wexlerae, Ruminococcus gnavus*	Rectal feces	Angus cattle and Xinjiang brown cattle		[34]
Backfat thickness in longissimus dorsi	Negative	Genus	*Blautia wexlerae*				
MUFA proportion	Negative	Phylum	Firmicutes	Rumen	Holstein bulls		[30]
n-3 PUFA proportion	Negative	Phylum	Saccharibacteria				
n-6 PUFA proportion	Positive	Genus	*Butyrivibrio_2*				
PUFA proportion	Positive	Genus	*Butyrivibrio_2, Ruminococcus_1*,				
	Negative	Genus	*Treponema_2*				
C18:0 proportion	Positive	Phylum	Firmicutes, Tenericutes				
	Negative	Phylum	*Proteobacteria*				
		Genus	*Prevotellaceae_UCG_004*				
Intramuscular fat	Positive	Family	unclassified [Mogibacteriaceae]	Feces	The unique multibreed Angus-Brahman herd with breed composition ranging from 100% Angus to 100% Brahman.		[35]
	Positive	Genus	*Succiniclasticum*				
The concentration of histidine in the longissimus dorsi	Positive	Genus	*Fibrobacter*	Rumen	Hu lambs	The effect of forage rape (Brassica napus)	[78]
	Negative		*Quinella*				
Intramuscular concentration of C20:3n6	Positive		*Schwartzia, Olsenella*				
Intramuscular concentration of C22:0	Negative		*Family_XIII_AD3011_group, Lachnospiraceae_FCS020_group*				
Intramuscular concentration of C20:4n6	Negative		*Family_XIII_AD3011_group, Lachnospiraceae_FCS020_group*				
Intramuscular concentration of C20:0	Negative		*Anaerovorax*				
Intramuscular concentration of C22:0	Negative		*Anaerovorax*				
Intramuscular concentration of C18:3n3	Negative		*Anaerovorax*				
Intramuscular fat in longissimus lumborum	Positive	Genus	*Fibrobacter, Succinivibrio*	Rumen	Ujimqin lambs	The effect of Alfalfa	[29]
Moisture in longissimus lumborum	Negative		*Fibrobacter, Succinivibrio*				
Palmitic (C16:0) in longissimus lumborum	Positive		*Fibrobacter, Succinivibrio*				
Stearic (C18:0) in longissimus lumborum	Positive		*Fibrobacter, Succinivibrio*				
Elaidic (C18:1 trans-9 in longissimus lumborum	Positive		*Fibrobacter, Succinivibrio*				
α-linolenic (C18:3n3) in longissimus lumborum							
	Positive		*Fibrobacter, Succinivibrio*				
n-6 PUFAs	Positive	Genus	*Selenomonas 1*	Rumen	Black Tibetan sheep’s	The effect of feed regimes	[31]
n-6/n-3 ratio	Positive		*Christensenellaceae R-7 group*				
	Negative		*Rikenellaceae RC9 gut group, Prevotella 1*				
n-3 PUFAs	Positive		*Lactobacillus*				
C16:0/C18:1 ratio	Positive		*Lactobacillus*				
	Negative		*Methanobrevibacter, Ruminococcus 2*				
n-3 PUFAs	Negative		*[Eubacterium] coprostanoligenes group, Quinella, Christensenellaceae R-7 group*				
C12:0 fatty acid content of longissimus muscle	Positive	Species	*Achromobacter xylosoxidans, Oscillibacter sp. PEA192, Mageeibacillus indolicus, Flavonifracter plautii*	Rumen	Tan sheep and Dorper sheep		[32]
	Negative		*Methanobrevibacter millerae*				
	Positive		*Mycobacterium dioxanotrophicus*	Duodenum			
	Negative		*Solibacillus silvestris, Advenella mimigardefordensis*				
	Negative		*Bacteroidales bacterium CF, Solitalea canadensis, Bacteroides coprosuis, Parabacteroides distasonis, Selenomonas ruminantium, Mucinivorans hirudinis, Paenibacillus sp. FSL H7-0737, Rhodococcus rhodochrous, Corynebacterium humireducens*	Colon			
C10:0 fatty acid content of longissimus muscle	Positive		*Achromobacter xylosoxidans*	Rumen			
	Negative		*Advenella mimigardefordensis*	Duodenum			
C14:1 fatty acid content of longissimus muscle	Positive		*Achromobacter xylosoxidans*	Rumen			
	Negative		*Advenella mimigardefordensis*	Duodenum			
C18:0 fatty acid content of longissimus muscle	Negative		*Advenella mimigardefordensis*	Duodenum			
C20:3n3 fatty acid content of longissimus muscle	Negative		*Pseudomonas stutzeri*	Colon			
